# Early orthodontic treatment need over a 10-year period and evaluation of short-term intervention stability

**DOI:** 10.1007/s00784-024-06104-4

**Published:** 2024-12-13

**Authors:** Maike Tabellion, Ines Caroline Loef, Constanze Charlotte Linsenmann, Jörg Alexander Lisson

**Affiliations:** Department of Orthodontics (G56), Saarland University, Kirrberger Strasse 100, 66424 Homburg/Saar, Germany

**Keywords:** Early orthodontic treatment, Orthodontic treatment need, Orthodontic indication groups, Treatment stability

## Abstract

**Objective:**

Early orthodontic treatment with cost reimbursement within the framework of the German statutory health insurance (GKV) is only possible for a strictly defined malocclusion group as defined by the orthodontic indication groups (KIG). It is not yet clear whether the application of the KIG criteria and corresponding successful early orthodontic interventions result in no or significantly less need for treatment in the late mixed dentition or in the permanent dentition. This study therefore investigated short-term intervention stability from a 10-year-period.

**Materials and methods:**

Between 2009 and 2019, *n* = 661 patients were diagnosed with indication groups D (increased overjet), M (reversed overjet), B (scissors bite), K (crossbite), or P (lack of space) including orthodontic treatment need. *N* = 70 patients (35 female, 35 male) met the inclusion criteria of the study and had received early orthodontic treatment with a mean duration of 15.44 ± 2.20 months. Orthodontic indication groups (KIG) were evaluated at the beginning (aged 7.99 ± 1.44 years) and the end of early orthodontic treatment (aged 9.63 ± 1.49 years) and at a voluntary control or the beginning of additional orthodontic treatment (aged 11.85 ± 1.72 years). The evaluation included established procedures for categorization of orthodontic indication groups and their respective classification. Statistics included Chi-square test and Kendall´s tau-b. The level of significance was set at *p* < 0.05.

**Results:**

The results showed reversed overjet in 44.3% and crossbites in 41.4% of the patients as most common indication for early orthodontic treatment. At the end of early orthodontic treatment, no orthodontic treatment need was present in 87.1%. At the late mixed dentition, the treatment result of early orthodontic treatment was stable in *N* = 61 out of *N* = 70 patients.

**Conclusions:**

The results of our study confirm preventive benefits of early orthodontic treatment, especially in patients with transverse anomalies or reversed overjet.

**Clinical relevance:**

A short-term orthodontic intervention with correct indication during primary or early mixed dentition can prevent or reduce further treatment need during late mixed or permanent dentition, and should therefore not be postponed.

## Introduction

In Germany, treatment of orthodontic anomalies and its costs covered by statutory health insurance (GKV), has been restricted by introduction of orthodontic indication groups (KIG) since January 2002 [8, 16, 23]. The KIG system categorizes orthodontic anomalies by eleven groups with special regard to etiology and five severity grades (Table 1). Orthodontists have to evaluate the indication group and grade during the first orthodontic appointment. Concerning the statutory health insurance, costs for orthodontic treatment are only defrayed for grades 3 to 5. Grades 1 and 2 are not covered by the health insurance, even if correction for medical reasons would be recommendable in individual cases [15, 23]. Differences of cost absorption occur, if early or main orthodontic treatment is needed. Early orthodontic treatment at the expense of the statutory health insurance is only possible for patients starting from age four with increased overjet (KIG D5), reversed overjet (KIG M4 and M5), scissors bite (KIG B4), bilateral or unilateral crossbite (KIG K3 and K4) and pronounced lack of space (KIG P3 and P4). Comprehensive orthodontic treatment at the expense of the statutory health insurance requires a late mixed dentition and is possible for patients aged under 18 years with cleft lip, alveolar process and/or palate and other craniofacial anomalies (KIG A5), hypodontia prior to prosthodontics or if space closure is indicated (KIG U4), eruption disorder with retention or displacement (KIG S4 and S5), increased overjet (KIG D4 and D5), negative overbite (KIG O3, O4 and O5), increased overbite (KIG T3), scissors bite (KIG B4), bilateral or unilateral crossbite (KIG K3 and K4), displacement of contact point (KIG E3 and E4) or lack of space (KIG P3 and P4). Instead of main orthodontic treatment, intervention at the expense of the health insurance is possible prior to a late mixed dentition for patients with KIG A5, M4 and M5 and O5. Combined orthodontic-surgical treatment at the expense of the statutory health insurance is possible for patients of any age after completion of growth with KIG A5, D4 and D5, M4 and M5, O5, B4 and K4 [15, 23]. The latest German Study on Oral Health (DMS • 6) evaluated the prevalence of malocclusion in 705 eight- to nine-year-olds and the need of orthodontic treatment with regard to KIG [11]. Since radiographs were not indicated as part of the study, indication groups U (Hypodontia) and S (eruption disorder) were excluded. Due to the nine remaining indication groups, orthodontic treatment need with grades 3 to 5 was found in 40.4% of the participants [11, 12, 16, 17]. Since the orthodontic indication groups (KIG) are used by orthodontists in Germany only, Kirschneck et al. [17] aimed to assess the Index of Orthodontic Treatment Need in a modified version (mIOTN) and the Index of Complexity, Outcome and Need (ICON) during the DMS 6 making international comparison easier. IOTN is established in Great Britain, ICON across Europe and the USA. Orthodontic treatment need was 40.4% using KIG, 41.6% using ICON and 44.2% using mIOTN. They confirmed that KIG can be used as a valid and reliable system to assess orthodontic treatment need without causing an over- or undersupply of orthodontic treatment in Germany.

**Table 1:** Orthodontic indication groups (KIG) for early (light blue) and main (≥ Grade 3) orthodontic treatment (distances in mm)

Indication group	Grade	1	2	3	4	5
Craniofacial Anomaly	A					Cleft lip/alveolar process/palate and other craniofacial anomalies
Hypodontia *Aplasia or tooth loss*	U				Hypodontia Prior to prosthodontics or space closure	
Eruption disorder	S				Retention (third molars excluded)	Displacement (third molars excluded)
Sagittal Anomaly *Increased overjet*	D	≤ 3.0	3.1 to 6.0		6.1 to 9.0	≥ 9.1
Sagittal Anomaly *Reversed overjet*	M				0.0 to 3.0	≥ 3.1
Vertical Anomaly *Negative overbite*	O	≤ 1.0	1.1 to 2.0	2.1 to 4.0	≥ 4.1 Habitual open bite	≥ 4.1 Skeletal open bite
Vertical Anomaly *Increased overbite*	T	≥ 1.1	≥ 3.1 With/without contact to gingiva	≥ 3.1 With traumatic contact to gingiva		
Transverse Anomaly *Scissors bite*	B				Scissors bite	
Transverse Anomaly *Crossbite*	K		End-to-end bite	Bilateral crossbite	Unilateral crossbite	
Displacement of contact point *Crowding*	E	≤ 1.0	1.1 to 3.0	3.1 to 5.0	≥ 5.0	
Lack of space	P		≤ 3.0	3.1 to 4.0	≥ 4.0	

The right timing of orthodontic treatment has been widely discussed especially with regard to immediate treatment effects and long-term benefits [10]. Early orthodontic treatment takes place in primary or early mixed dentition and aims to correct dental and skeletal anomalies as early as possible in terms of proceeding growth. After early orthodontic treatment and reduced severity of malocclusion, later main orthodontic treatment is either not needed anymore or easier and shorter with more stable results in the long term [2, 10, 14, 19, 21, 24]. Especially unfavorable growth and underdeveloped jaws combined with dental anomalies can be influenced by functional orthodontic and orthopedic appliances at an early age [10]. Long-term stability of early orthodontic treatment is also widely and controversially discussed [7, 25]. Dolce et al. [7] reported in their centrographic analysis of 1-phase or 2-phase treatment for class II malocclusion that early treatment has effects on the mandible, but the effects were not apparent at the end of fixed appliance treatment. Tulloch et al. [25] suggested in their randomized clinical trial of early class II treatment that a 2-phase treatment started early before adolescence might be not more effective than a 1-phase treatment during adolescence in the permanent dentition. Many orthodontists postpone orthodontic treatment until complete eruption of the permanent dentition and believe that there is no significant difference of an early or late initiated treatment [10].

### Aims of the study

The aim of this study was to investigate whether there is a new or recurring need for orthodontic treatment in the short-term after successful early orthodontic treatment in relation to the orthodontic indication groups (KIG). It should be verified, if the treatment results of early orthodontic treatment are preventive with special regard to later main orthodontic treatment need.

## Materials and methods

### Patients

Between 2009 and 2019, *n* = 661 patients diagnosed by means of indication groups with classification grades D5, M5, M4, B4, K4, K3, P4 or P3 for orthodontic treatment at Saarland University Hospital were identified using the dental software ivoris (Computer konkret AG, Falkenstein, Germany). Out of those, 70 patients came back for a check-up after early orthodontic treatment and 64 patients had ongoing early orthodontic treatment.

### Inclusion/exclusion criteria

Inclusion criteria were completed early orthodontic treatment and diagnostic data including dental casts at the beginning (t_0_, aged 7.99 ± 1.44 years) and at the end of early orthodontic treatment (t_1_, aged 9.63 ± 1.49 years) and dental casts and a panoramic x-ray at a check-up or rather the beginning of main orthodontic treatment (t_2_, aged 11.85 ± 1.72 years).

Exclusion criteria included cleft lip and/or palate, comorbid syndromes and genetic disorders.

Out of the *n* = 661 patients, *n* = 70 patients (35 female, 35 male) met the inclusion criteria of the study. All 70 patients were exclusively treated at the Saarland University Hospital. Mean duration of early orthodontic treatment was 15.44 ± 2.20 months.

### Cast and orthopantogram evaluation

A total of 210 paired casts and 70 orthopantograms of patients from one specialized center were available with 70 paired casts for each point in time. Fabrication of dental casts needed alginate impressions (Kaniblue, Kanidenta GmbH & Co. KG, Herford, Germany) and they were made of type III hard plaster (Hinritzit^®^, Ernst Hinrichs, Goslar, Germany). The casts of t_0_, t_1_ and t_2_ were evaluated concerning overjet, overbite, displacement of contact point or lack of space with reagard to the orthodontic indication groups by the use of a sliding caliper »Münchner Modell^®^« (Dentaurum, Ispringen, Germany) or visually concerning transverse anomalies by one orthodontist. If needed, the greatest tooth aberrance was measured in millimeters with a precision of 0.25 mm. The orthopantograms of t_2_ were evaluated visually with special regard to hypodontia or eruption disorders. Craniofacial anomalies were diagnosed visually or based on documentation of genetic screenings.

### Statistical method, error of the method

Statistical analysis was performed with the SPSS software version 26 (IBM, Armonk, NY, USA). Statistics included chi-square test for differences concerning orthodontic indication groups of female and male patients at each point in time and between the different points in time of overall patients. The level of significance was set at *p* < 0.05. The significance level was defined as follows: *p* ≥ 0.05 not significant, *p* < 0.05 significant, *p* < 0.01 highly significant and *p* < 0.001 most highly significant. Chi-square test used for differences concerning orthodontic indication groups between the different points in time of overall patients was followed by testing the correlation using Kendall´s tau-b (τb): 0.1 = small effect size and low correlation, 0.3 = medium effect size and correlation, 0.5 = large effect size and high correlation. For testing the interrater-reliability the whole evaluation process was repeated by a second orthodontist two months after the first investigation. Intrarater-reliability was not tested to reduce any degree of subjectivity. The differences were statistically analyzed using again Kendall´s tau-b (τb). Correlation τb was 1.000 at t_0_, 0.728 at t_1_ and 0.876 at t_2_. According to power analysis using the software G*Power version 3.1.9 (HHU, Düsseldorf, Germany), with an effect size of 0.50 and an alpha level of 0.05 the actual power was 91.5%.

## Results

### Indication groups and grades at t0 for all 70 patients (Fig. 1; Table 2)

**Table 2:** Indication groups and grades at t_0_ for all 70 patients and divided into female and male patients

Indication group and grade	Overall patients		Female patients		Male patients	
	Quantity	[%]	Quantity	[%]	Quantity	[%]
P3	2	2.9	1	1.4	1	1.4
P4	1	1.4	0	0.0	1	1.4
K3	4	5.7	2	2.9	2	2.9
K4	25	35.7	11	15.7	14	20.0
B4	2	2.9	0	0.0	2	2.9
M4	30	42.9	18	25.7	12	17.1
M5	1	1.4	0	0.0	1	1.4
D5	5	7.1	3	4.3	2	2.9

**Fig. 1:**
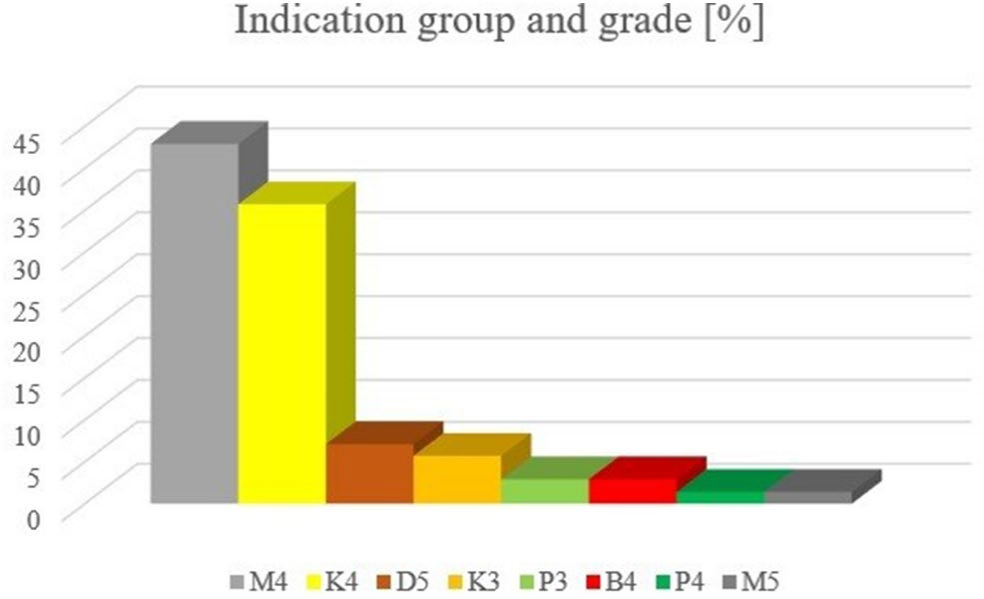
Percentage of indication group and grade of *n* = 70 patients at t_0_

At the beginning of early orthodontic treatment (t_0_) reversed overjet (M4) was present in *n* = 30 patients (42.9%) and unilateral crossbite (K4) in *n* = 25 patients (35.7%). *N* = 5 patients (7.1%) presented increased overjet (D5) and *n* = 4 patients (5.7%) bilateral crossbite (K3). Lack of space (P3) and scissors bite (B4) were equally present in *n* = 2 patients (2.9%) respectively. Lack of space (P4) and reversed overjet (M5) were equally present in *n* = 1 patient (1.4%) each. The differences of the distribution between indication groups and grades of female and male patients at t_0_ were not significant (*p* = 0.568).

### Indication groups and grades at t1 for all 70 patients (Fig. 2; Table 3)

**Table 3:** Indication groups and grades at t_1_ for all 70 patients and divided into female and male patients

Indication group and grade	Overall patients		Female patients		Male patients	
	Quantity	[%]	Quantity	[%]	Quantity	[%]
No treatment need	61	87.1	29	41.4	32	45.7
P3	1	1.4	0	0	1	1.4
P4	5	7.1	4	5.7	1	1.4
M4	1	1.4	0	0	1	1.4
D4	2	2.9	2	2.9	0	0.0

**Fig. 2:**
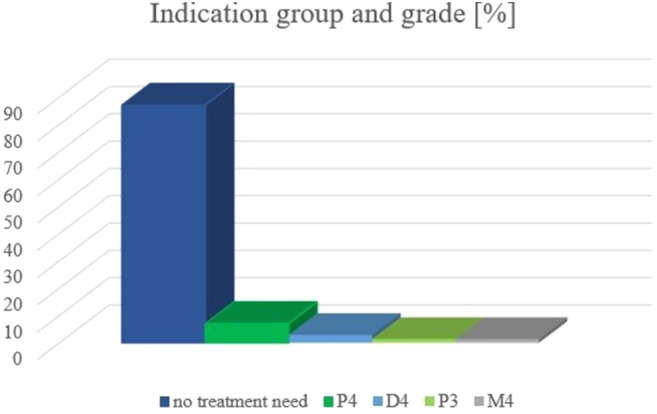
Percentage of indication group and grade of *n* = 70 patients at t_1_

At the end of early orthodontic treatment (t_1_) no further treatment need was present in *n* = 60 patients (87.1%). *N* = 5 patients (7.1%) presented lack of space (P4) and *n* = 2 patients (2.9%) increased overjet (D4). Lack of space (P3) and reversed overjet (M4) were equally present in *n* = 1 patient (1.4%) respectively. The differences of the distribution between indication groups and grades of female and male patients at t_1_ were not significant (*p* = 0.203).

### Indication groups and grades at t2 for all 70 patients (Fig. 3; Table 4)

**Table 4:** Indication groups and grades at t_2_ for all 70 patients and divided into female and male patients

Indication group and grade	Overall patients		Female patients		Male patients	
	Quantity	[%]	Quantity	[%]	Quantity	[%]
No treatment need	16	22.9	10	14.3	6	8.6
P4	1	1.4	0	0.0	1	1.4
E3	6	8.6	3	4.3	3	4.3
E4	3	4.3	1	1.4	2	2.9
K4	5	7.1	2	2.9	3	4.3
B4	2	2.9	1	1.4	1	1.4
T3	2	2.9	0	0.0	2	2.9
M4	12	17.1	3	4.3	9	12.9
D4	8	11.4	3	4.3	5	7.1
S4	10	14.3	8	11.4	2	2.9
S5	4	5.7	3	4.3	1	1.4
U4	1	1.4	1	1.4	0	0.0

**Fig. 3:**
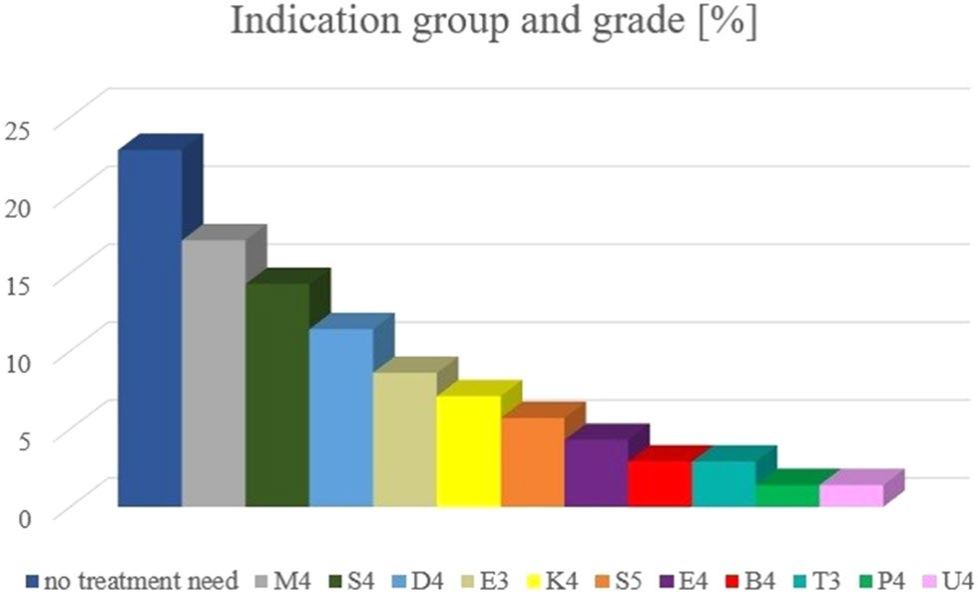
Percentage of indication group and grade of *n* = 70 patients at t_2_

At the beginning of main orthodontic treatment (t_2_), the treatment result of early orthodontic treatment was stable in *N* = 61 out of *N* = 70 patients (87.1%). *N* = 12 patients (17.1%) presented reversed overjet (M4) and *n* = 10 patients (14.3%) retention of teeth (S4). *N* = 8 patients (11.4%) presented increased overjet (D4) and *n* = 6 patients (8.6%) displacement of contact point (E3). *N* = 5 patients (7.1%) presented unilateral crossbite (K4) and *n* = 4 patients (5.7%) displacement of teeth (S5). Displacement of contact point (E4) was present in *n* = 3 patients (4.3%). Scissors bite (B4) and increased overbite (T3) were equally present in *n* = 2 patients (2.9%) respectively. Lack of space (P4) and hypodontia (U4) were equally present in *n* = 1 patient (1.4%) each. The differences of the distribution between indication groups and grades of female and male patients at t_2_ were not significant (*p* = 0.254).

### Change of indication group and grade between t0 and t2 for all 70 patients (Fig. 4; Tables 5 and 8

**Table 5:** Change of indication group and grade between t_0_ (vertical column) and t_2_ (horizontal column) for all 70 patients

Indication group and grade	No treatment need	P4	E3	E4	K4	B4	T3	M4	D4	S4	S5	U4	Overall patients
P3			2										2
P4				1									1
K3								3		1			4
K4	8			1	3	1	1	4	3	4			25
B4						1	1						2
M4	8		4	1	2			5	2	4	3	1	30
M5											1		1
D5		1							3	1			5

**Table 6:** Kendall´s tau-b (τb) correlation of indication groups and grades between t_0_, t_1_ and t_2_ for all 70 patients

	Indication group and grade t_1_	Indication group and grade t_2_
**Indication group and grade t**_**0**_	0.072	0.116
*P value* ^*a*^	0.522	0.233
**Indication group and grade t**_**1**_		0.119
*P value* ^*a*^		0.254

^a^Chi-square test

**Fig. 4:**
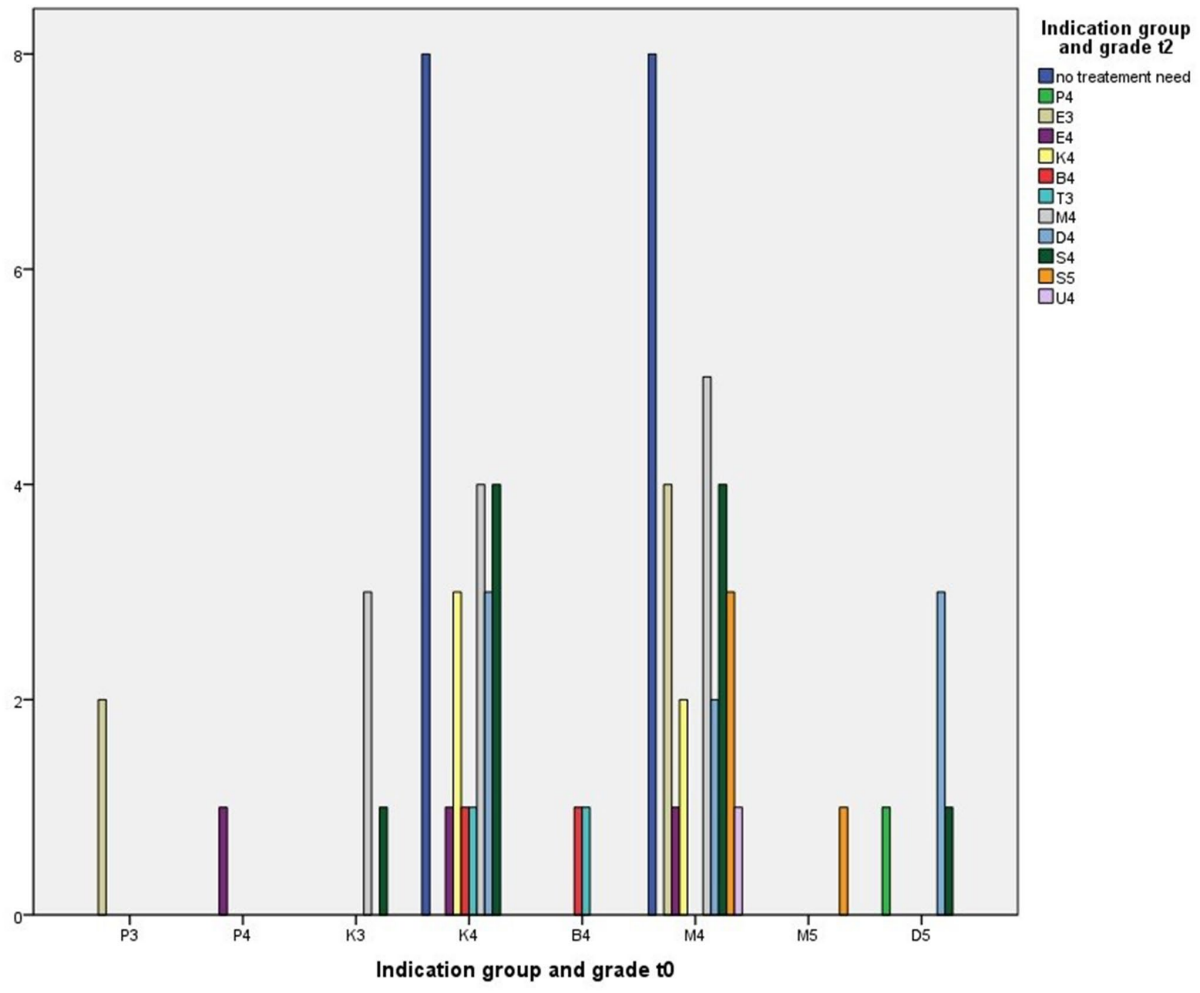
Change of indication group and grade of *n* = 70 patients between t_0_ and t_2_

*N* = 2 out of *n* = 2 patients with lack of space (P3) at t_0_ presented displacement of contact point (E3) at t_2_. *N* = 1 out of *n* = 1 patient with lack of space (P4) at t_0_ presented displacement of contact point (E4) at t_2_. Out of *n* = 4 patients with bilateral crossbite (K3) at t_0_*n* = 3 patients presented reversed overjet (M4) and *n* = 1 patient retention of teeth (S4) at t_2_. Out of *n* = 25 patients with unilateral crossbite (K4) at t_0_*n* = 8 patients presented no treatment need, *n* = 1 patient displacement of contact point (E4), *n* = 1 patient scissors bite (B4), *n* = 1 patient increased overbite (T3), *n* = 4 patients reversed overjet (M4), *n* = 3 patients increased overjet (D4), *n* = 4 patients retention of teeth (S4) and in *n* = 3 patients unilateral crossbite (K4) recurred at t_2_. Out of *n* = 2 patients with scissors bite (B4) at t_0_*n* = 1 patient presented increased overbite (T3) and in *n* = 1 patient scissors bite (B4) recurred at t_2_. Out of *n* = 30 patients with reversed overjet (M4) at t_0_*n* = 8 patients presented no treatment need, *n* = 4 patients displacement of contact point (E3), *n* = 1 patient displacement of contact point (E4), *n* = 2 patients unilateral crossbite (K4), *n* = 2 patients increased overjet (D4), *n* = 4 patients retention of teeth (S4), *n* = 3 patients displacement of teeth (S5), *n* = 1 patient hypodontia (U4) and in *n* = 5 patients reversed overjet (M4) recurred at t_2_. *N* = 1 patient with reversed overjet (M5) at t_0_ presented displacement of teeth (S5) at t_2_. Out of *n* = 5 patients with increased overjet (D5) at t_0_*n* = 1 patient presented lack of space (P4), *n* = 3 patients less, but still increased overjet (D4) and *n* = 1 patient retention of teeth (S4) at t_2_. The changes of indication groups and grades in terms of correlation of overall patients between t_0_ and t_2_ were not significant (*p* = 0.233, τb = 0.116).

### Change of indication group and grade between t0 and t1 for all 70 patients (Tables 6 and 8)

*N* = 2 out of *n* = 2 patients with lack of space (P3) at t_0_ presented no treatment need at t_1_. *N* = 1 out of *n* = 1 patient with lack of space (P4) at t_0_ presented no treatment need at t_1_. *N* = 4 out of *n* = 4 patients with bilateral crossbite (K3) at t_0_ presented no treatment need at t_1_. Out of *n* = 25 patients with unilateral crossbite (K4) at t_0_*n* = 20 patients presented no treatment need, *n* = 3 patients lack of space (P4), *n* = 1 patient reversed overjet (M4) and *n* = 1 patient increased overjet (D4) at t_1_. *N* = 2 out of *n* = 2 patients with scissors bite (B4) at t_0_ presented no treatment need at t_1_. Out of *n* = 30 patients with reversed overjet (M4) at t_0_*n* = 28 patients presented no treatment need and *n* = 2 patients lack of space (P4) at t_1_. *N* = 1 out of *n* = 1 patient with reversed overjet (M5) at t_0_ presented no treatment need at t_1_. Out of *n* = 5 patients with increased overjet (D5) at t_0_*n* = 3 patients presented no treatment need, *n* = 1 patient lack of space (P3) and *n* = 1 patient less, but still increased overjet (D4) at t_1_. The changes of indication groups and grades in terms of correlation of overall patients between t_0_ and t_1_ were not significant (*p* = 0.522, τb = 0.072).

### Change of indication group and grade between t1 and t2 for all 70 patients (Tables 7 and 8)

**Table 7:** Change of indication group and grade between t_1_ (vertical column) and t_2_ (horizontal column) for all 70 patients

Indication group and grade	No treatment need	P4	E3	E4	K4	B4	T3	M4	D4	S4	S5	U4	Overall patients
No treatment need	16		6	3	5	2	2	11	6	6	3	1	61
P3		1											1
P4										4	1		5
M4								1					1
D4									2				2

**Table 8:** Change of indication group and grade between t_0_ (vertical column) and t_1_ (horizontal column) for all 70 patients

Indication group and grade	No treatment need	P3	P4	M4	D4	Overall patients
P3	2					2
P4	1					1
K3	4					4
K4	20		3	1	1	25
B4	2					2
M4	28		2			30
M5	1					1
D5	3	1			1	5

Out of *n* = 61 patients with no treatment need at t_1_*n* = 16 patients presented still no treatment need, *n* = 6 patients displacement of contact point (E3), *n* = 3 patients displacement of contact point (E4), *n* = 5 patients unilateral crossbite (K4), *n* = 2 patients scissors bite (B4), n = s patients increased overbite (T3), *n* = 11 patients reversed overjet (M4), *n* = 6 patients increased overjet (D4), *n* = 6 patients retention of teeth (S4), *n* = 3 patients displacement of teeth (S5) and *n* = 1 patients hypodontia (U4) at t_2_. *N* = 1 out of *n* = 1 patient with lack of space (P3) at t_1_ presented more lack of space (P4) at t_2_. Out of *n* = 5 patients with lack of space (P4) at t_1_*n* = 4 patients presented retention of teeth (S4) and *n* = 1 patient displacement of teeth (S5) at t_2_. *N* = 1 out of *n* = 1 patient with reversed overjet (M4) at t_1_ presented again reversed overjet (M4) at t_2_. *N* = 2 out of *n* = 2 patients with increased overjet (D4) at t_1_ presented again increased overjet (D4) at t_2_. The changes of indication groups and grades in terms of correlation of overall patients between t_1_ and t_2_ were not significant (*p* = 0.254, τb = 0.119).

## Discussion

In our study, early orthodontic treatment (aged 7.99 ± 1.44 years) was most frequently indicated because of reversed overjet (M4 and M5) in 44.3% of the patients, uni- and bilateral crossbites (K4 and K3) in 41.4% of the patients and increased overjet (D5) in 7.1% of the patients. At the end of early orthodontic treatment (aged 9.63 ± 1.49 years) 87.1% of the patients had no further treatment need. Two years later (aged 11.85 ± 1.72 years), 22.9% of the patients still had no need for main orthodontic treatment at the late mixed dentition stage. No further treatment costs for main orthodontic treatment within the framework of the statutory health insurance were incurred. Therefore, early orthodontic treatment prevented main orthodontic treatment need in 16 patients of our study successfully. 54 patients had main orthodontic treatment need. 45 patients of this sample presented new indication groups and grades for main orthodontic treatment need, the treatment results of early orthodontic treatment were stable. 9 patients presented the same indication group and grade for main orthodontic treatment need as they had for early orthodontic treatment need. Against ongoing debates concerning benefits of early orthodontic treatment, there are many national and international studies advocating early intervention [1, 3–6, 13, 18, 20]. Almeida et al. [1] presented in their case report a twelve-year-old girl with posterior crossbite correction. The treatment result of the posterior crossbite correction was stable even after 21 years of following-up. Bartzela and Jonas [3] described observation periods after unilateral posterior crossbite correction of 8 years for an early treatment group and 6.5 years for the late treatment group. In almost 80% of the patient the result of the posterior crossbite correction remained stable in long-term. The short-term stability of unilateral posterior crossbite treatment was 75% in our study. Defraia et al. [6] compared 23 patients treated with a removable appliance for unilateral posterior crossbite treatment during primary or early mixed dentition with 20 untreated patients. Positive dental and skeletal effects of the treatment were seen in short-term after 22 months compared to the untreated control. Kerosuo et al. [13] followed-up 68 patients between 8 and 20 years, who have been treated after the first examination at 8 years of age due to anterior or lateral crossbite, increased overjet or overbite or severe crowding. They suggested, that definite orthodontic treatment need may be eliminated by early treatment with simple appliances and that early timing of treatment may have led to good long-term stability of the treatment results. 78% of the patients had no treatment need at the age of 20.

Especially early-corrected overjet and crossbite tend to be stable in long-term [3, 13]. In our study, main orthodontic treatment was most frequently indicated because of retention and displacement of teeth (S4 and S5) in 25.9% of the 54 patients with further treatment need followed by reversed overjet (M4) in 17.1% of the patients and increased overjet (D4) in 11.4% of the patients. Correlation tends to exist between transverse anomalies (K3 and K4) and later retention of teeth (S4) and between reversed overjet (M4 and M5) and later displacement of contact point (E3 and E4) or retention or displacement of teeth (S4 and S5). Nonetheless, correlations were not significant in our study.

To our knowledge, our study is the first presenting longitudinal data related to orthodontic indication groups by comparison of early and main orthodontic treatment need and short-term stability of early orthodontic treatment results taking the changes of KIG into account. Graf et al. [9] screened 586 patients from seven German study centers for their cohort study and rated the severity of malocclusion using the Peer Assessment Rating (PAR)-Index [22] providing an objective assessment of treatment success at baseline and after retention period. Their study comprised 335 patients, mean age 14.8 years. 164 patients improved greatly during treatment. 81.5% of all patients had a high-quality treatment outcome.

### Limitations of the study

The number of patients of our study was acceptable. Nonetheless, a larger patient number could have been reached, but 53 patients did not return for a recall after completion early orthodontic treatment, 20 patients returned for a check-up after early orthodontic treatment, but dental casts for evaluation were not made and 13 patients cancelled the early orthodontic treatment before completion. 50 patients with cleft lip and/or palate, comorbid syndromes or genetic disorders were excluded from the study. Due to the small number of patients, a gender division was not performed during comparison of treatment results and short-term stability. The recruitment duration was rather long, since only a few patients are referred for early orthodontic treatment during primary or early mixed dentition. Most of our patients were referred for treatment at a later age, because the dentists told them to postpone orthodontic treatment until permanent dentition. In addition, the orthodontic indication groups (KIG) are used by orthodontists in Germany only, therefore international comparability is difficult.

Finally, treatment success and resulting short-term stability does not exclusively depend on correct indication and timing, but also on patient and parents compliance, especially when removables are used.

## Conclusion

The results of our study confirm preventive benefits of early orthodontic treatment, especially in patients with transverse anomalies or reversed overjet. At the end of early orthodontic treatment, no orthodontic treatment need was present in 87.1%. At the late mixed dentition, the treatment result of early orthodontic treatment was stable in *N* = 61 out of *N* = 70 patients. A short-term orthodontic intervention with correct indication during primary or early mixed dentition can prevent or reduce further treatment need during late mixed or permanent dentition, and should therefore not be postponed.

## Data Availability

No datasets were generated or analysed during the current study.
